# The use of whole genome sequencing to study young patients with 100+ adenomas of the colon

**DOI:** 10.3389/fonc.2025.1616441

**Published:** 2025-10-15

**Authors:** Aleksey S. Tsukanov, Sergey I. Achkasov, Anna N. Loginova, Vitaly P. Shubin, Dmitry Y. Pikunov, Nikolay N. Chekanov, Anastasiya S. Salomatina, Konstantin V. Severinov

**Affiliations:** ^1^ Ryzhikh National Medical Research Centre for Coloproctology, Moscow, Russia; ^2^ ”Biotechnology campus” LLC, Moscow, Russia

**Keywords:** familial adenomatous polyposis, APC, BMPR1A, juvenile polyposis syndrome, whole genome sequencing

## Abstract

**Objective:**

Adenomatous polyposis syndrome (APS) is a rare hereditary disease characterized by the development of multiple (more than 20) adenomas of the colon with a high-risk of malignant transformation without surgical treatment. The most aggressive form of APS, with >100 polyps before the age of 45 years, is mostly caused by germline pathogenic variants in the *APC* gene but patients with germline variants in the *MUTYH* and, very rarely, in the *SMAD4* and *BMPR1A* genes were also reported. Routine molecular testing methods, such as Sanger sequencing, multiplex ligation-dependent probe amplification (MLPA) or multigene NGS panels, may fail to detect pathogenic variants in non-coding regions.

**Patients and methods:**

DNA from blood samples of 10 patients (with age of APS manifestation between 15 and 45 years) with over 100 adenomatous colonic polyps identified by endoscopic examination was subjected to whole genome sequencing (WGS). Prior genetic testing did not detect any germline pathogenic variants in the *APC* and *MUTYH* coding exons in these patients.

**Results:**

Pathogenic and likely pathogenic germline variants in non-coding regions of genes were identified in 3 patients. Two unrelated patients had the same c.-190G>A (rs879253785) in the 1B promoter of the *APC* gene (NM_001127511.3), while the third patient had a c.-152-2A>G variant in the *BMPR1A* gene (NM_004329.3). Using standard NGS panels or whole exome sequencing (WES) would not have detected these variants.

**Conclusion:**

Our results demonstrate that WGS is a useful genetic testing method for young patients with over 100 adenomatous colonic polyps, when routine DNA diagnostic methods fail to establish the genetic cause of the disease.

## Introduction

The most common form of adenomatous polyposis syndrome is familial adenomatous polyposis (FAP), (OMIM # 175100) - a hereditary syndrome characterized by dozens to hundreds (and even thousands) of colonic polyps and extremely high risk of colorectal cancer unless the polyps are detected and surgically removed early. The incidence of FAP is between 1 per 5000 and 1 per 18000 cases (1). The disease comes in attenuated (less than 100 polyps in patients aged above 45 years old) or classic (over 100 polyps in patients under 45 years old) forms, with the latter being more prevalent.

Both forms of FAP are mostly caused by pathogenic or likely pathogenic variants in the *APC* gene (OMIM # 611731). This gene encodes a protein involved in the WNT signaling pathway. According to ongoing studies in different countries, inherited *APC* variants in patients with over 100 colonic polyps are identified in 70-90% of cases (2). Detecting the pathogenic *APC* variants is essential as it confirms the diagnosis of classic FAP, which is an indication for the expanded surgical intervention, i.e., proctocolectomy (3).

Apart from the *APC* variants, adenomatous polyposis syndrome can be caused by biallelic variants in the *MUTYH* gene (OMIM # 604933). These patients are usually diagnosed with about 50 colonic polyps, though some can develop over 100 polyps (4). Very rarely, mutations in the *BMPR1A* (OMIM # 601299) and *SMAD4* genes (OMIM # 600993) can also cause development of the disease. Most pathogenic and likely pathogenic heterozygous variants in these genes cause the juvenile polyposis syndrome (JPS) (5). However, some JPS patients demonstrate both juvenile and adenomatous polyps (6). Furthermore, some authors report patients with *BMPR1A* and *SMAD4* mutations diagnosed only with adenomatous hyperplasia (7).

This study describes the results of WGS testing of ten Russian patients with over 100 adenomatous colonic polyps who had no pathogenic variants identified by routine DNA testing methods (Sanger sequencing and MLPA).

## Patients and methods

Patients enrolled in the study included 6 men and 4 women (age of FAP manifestation between 15 and 45 years) and underwent treatment or observation at the Ryzhikh National Medical Research Centre for Coloproctology (RNMRCC), Ministry of Health of the Russian Federation from March 2020 to September 2023. Genetic testing of patients’ DNA from blood samples, performed prior to enrollment did not reveal any pathogenic or likely pathogenic *APC* and *MUTYH* variants by Sanger sequencing, MLPA. Informed consent was obtained from all patients enrolled in the study, which complied with the ethical principles of the Declaration of Helsinki and was approved by the Ethics Committee at RNMRCC.

## Whole genome sequencing, annotation and interpretation

Genomic DNA was extracted using the magnetic bead-based sorption method (MGIEasy Magnetic Beads Blood Genomic DNA Extraction Kit, MGI) and subsequently used for the preparation of genomic libraries for sequencing. Library preparation was performed using a PCR-free protocol with enzymatic DNA fragmentation (MGIEasy FS PCR-Free Library Prep Set, 96 reactions (MIX), MGI). Prepared libraries were sequenced on the DNBSEQ-T7 sequencer (PE150) with a target median coverage of 30×. Reads were passed to cutadapt 4.2 (8) for adapter removal (MGIEasy DNA Adapters) and trimming of low-quality read ends. Mapping to the GDC reference genome GRCh38.d1.vd1 was performed with bwa 0.7.17 (9). Duplicate marking was performed with Picard 2.27.5. Short variation calling was performed with DeepVariant 1.4 (10).

Variant annotation was performed with the Ensembl Variant Predictor (VEP (11),), including information from ClinVar 20240603 (12) and gnomAD v.4.1 (13) databases. SpliceAI (14) and Pangolin (15) were used for the estimation of variant effects on splice sites. PolyPhen-2 (16), CADD v.1.7 (17), and AlphaMissense (18) were used for the assessment of the pathogenicity of novel identified missense variants. Clinical significance interpretation of detected variants was performed in accordance with the ACMG guidelines (19).

Variants of clinical interest were confirmed by Sanger sequencing on ABI PRISM 3500 genetic analyzer (Applied Biosystems, USA) according to the manufacturer’s protocol.

## Results

WGS of DNA samples from 10 patients with over 100 adenomatous colonic polyps revealed pathogenic or likely pathogenic variants in 3 cases (**Table 1**). In all 3 cases, the findings were confirmed by Sanger sequencing.

**Table 1:** Clinical and relevant WGS data of patients with FAP.

№ pts	Gender	Age of manifestation	Number of polyps	Presence of colorectal cancer	Number of relatives with FAP	WGS
1	f	15	100+	-	0	*BMPR1A* (NM_004329.3): c.-152-2A>G p.?
2	m	39	100+	–	0	
3	m	20	100+	–	4	
4	f	30	1000+	–	2	*APC* (NM_001127511.3): c.-190G>A
5	f	15	1000+	–	3	
6	m	31	1000+	–	2	
7	m	37	100+	+	0	
8	f	45	100+	–	3	
9	m	41	1000+	+	unknown	*APC* (NM_001127511.3): c.-190G>A
10	m	26	200+	–	4	

**Figure 1:**
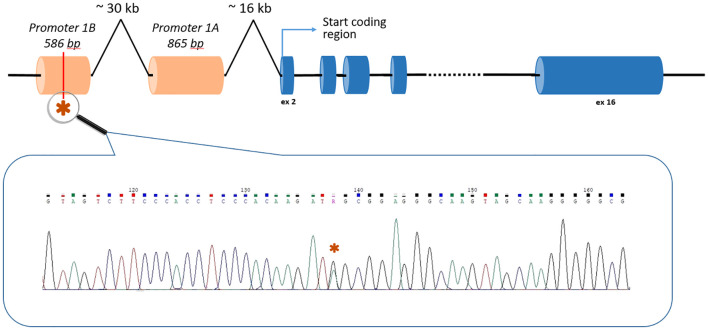
The position of the c.-190G>A *APC* variant in the context of the gene structure.

Two unrelated patients carried the c.-190G>A (rs879253785) variant in the Ying Yang 1 (YY1) binding motif of the 1B promoter of the *APC* gene (NM_001127511.3) (**Figure 1**). Li et al. (2016) showed that this variant reduced transcription from the 1B promoter by interfering with transcription factor YY1 binding. The authors performed segregation analysis and found that this variant was present in 5 affected relatives in 3 generations but not in healthy family members (20).

The first patient with the variant in the *APC* gene, a female aged 30, had over 1000 adenomatous colonic polyps ranging from 0.2 and 1.0 cm in size, which was an indication for restorative proctocolectomy. Pathological post-operative examination showed no malignant disease. According to family history, her mother died at the age of 33 from sigmoid cancer with dissemination, while her maternal grandmother died at the age of 45 from colon cancer.

The second patient with the variant in the *APC* gene, aged 41, also had over 1000 adenomatous polyps ranging from 0.1 and 0.4 cm and rectal cancer pT1N0M0 and underwent restorative proctocolectomy. The family history of the disease is unknown since the patient was an orphan.

**Figure 2:**
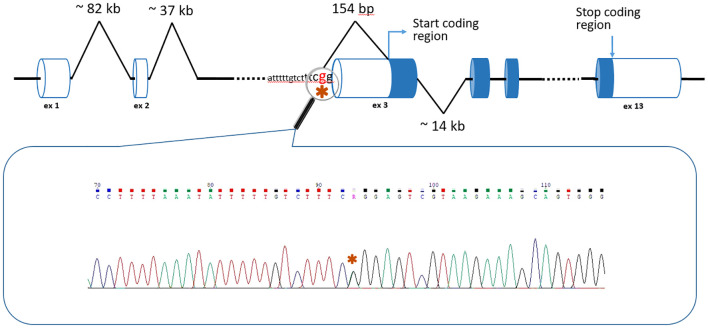
The position of the c.-152-2A>G *BMPR1A* variant (*) in the context of the gene structure.

In the third patient, a 24 year old female, WGS unexpectedly detected a c.-152-2A>G variant in the *BMPR1A* gene (NM_004329.3) (**Figure 2**). Thе variant has no reported population frequency in gnomAD (0 out of 152,316 alleles). The substitution affects the canonical splice acceptor site and is a loss-of-function variant in the gene where loss of function is a known mechanism of disease. The variant was classified as likely pathogenic (meeting PVS1, PM2 criteria) in accordance with the ACMG recommendations. To our knowledge, this variant was encountered only once and was described as pathogenic by Staninova-Stojovska et al. (2019) in a patient with over 100 juvenile polyps (21). According to medical history, our patient underwent multiple colon polypectomies, starting from 15 years of age. Over the years, she developed more than 100 polyps with maximal size up to 2.5 cm. She also underwent restorative proctocolectomy. The pathological post-operative examination showed no malignant disease and all investigated polyps were adenomas. Both of her parents are alive and healthy.

## Discussion

In our previous work, the frequency of pathogenic variants in the *APC* gene in a cohort of 108 patients with suspected FAP reached 72.2% (22). In that study, genetic testing of the *APC* coding exons was mostly carried out using conformation sensitive gel electrophoresis (CSGE) and Sanger sequencing was performed only for DNA fragments with altered mobility According to Kerr et al., who also employed, the frequency of pathogenic and likely pathogenic *APC* variants among 1591 patients with suspected FAP was only 27% (23). While CSGE allows successful detection of small insertions and deletions, it occasionally fails to identify single nucleotide variants. This made us start using Sanger sequencing for all *APC* coding exons as a routine genetic testing method for patients with suspected FAP. We also use MLPA to search for large *APC* deletions or duplications in patients without point mutations. The combination of these two methods allowed us to increase the detection rate of pathogenic (or likely pathogenic) *APC* variants in patients with classic FAP to 91.6% (24).

Since shifting to combined Sanger sequencing and MLPA did not allow us to establish genetic causes of the disease in all cases, we here assessed the use of WGS to further increase the diagnostic efficiency. WGS analysis of 10 FAP cases that had negative results on the Sanger/MLPA combination identified the previously described variant c.-190G>A in the *APC* gene in two patients and an extremely rare likely pathogenic variant c.-152-2A>G in the *BMPR1A* gene in one patient. Overall, inclusion of WGS allowed to increase the total incidence of identified variants in patients clinically diagnosed with classic FAP to over 94%.

The c.-190G>A (rs879253785) variant in the 1B promoter of the *APC* gene, which was detected in two unrelated patients with polyposis, is located at a distance of 47,363 bp from the first coding nucleotide of the gene, located in the second exon (**Figure 1**). Accordingly, none of the current standard NGS panels has the ability to detect it.

The situation with the c.-152-2A>G variant in the *BMPR1A* gene is more complicated. It is located only two nucleotides before the exon 3 of the gene, but the distance to the first coding nucleotide is 154 bp (**Figure 2**). Interestingly, the authors who previously discovered this variant used the AmpliSeq Designer custom panel (Life Technologies) for the Ion Torrent PGM sequencer (21). At the same time, it should be noted that amplicon panels have a number of significant limitations: heterogeneity of coverage, difficulties in detecting CNVs, but the most significant disadvantage is the small number of genes studied (for example, the custom panel of Staninova-Stojovska M. et al. (21) included only 15 genes, and it did not contain genes of some polyposis syndromes: *RNF43, NTHL1, MSH3*, etc.).

**Figure 3:**
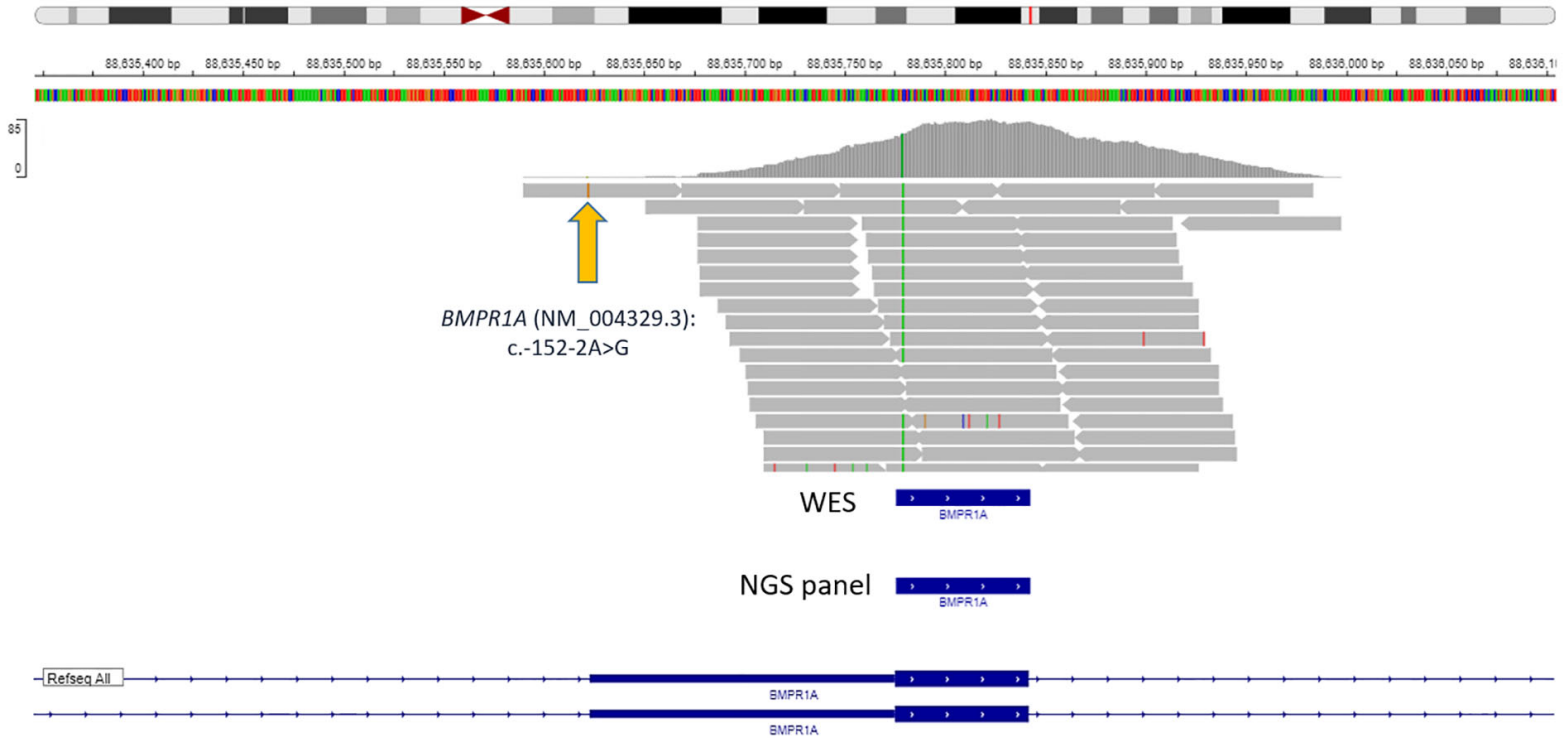
Data visualization of VANTS CORE Exome approach with variant *BMPR1A* (NM_004329.3): c.-152-2A>G.

We decided to find out whether modern standard NGS panels for studying hereditary cancers can detect this variant. First of all, we analyzed one of the largest oncopanels—NanOnco Plus Panel v3.0—which lists 637 genes, including *BMPR1A*. The c.-152-2A>G variant has coordinates 10-86875865-A-G (GRCh38), and the *BMPR1A* gene coverage in the panel starts with 10-86875981 (probe)/10-86876018 (target) according to the manufacturer’s documentation, respectively, thus it will not be able to detect this variant (**Figure 3**). Moreover, in our clinical practice we use WES to study the DNA of patients with hereditary forms of colorectal cancer, but it turned out that the c._152-2A>G variant also cannot be detected with WES (**Figure 3**).

These facts confirm the relevance of our decision to examine all 10 samples using the WGS method.

In the work of Staninova-Stojovska M. et al. in 2019 (21) the c.-152-2A>G variant in *BMPR1A* was described as pathogenic. It is important to note that the SpliceAI showed a 0.99 probability of acceptor loss and a 0.89 probability of donor loss; Pangolin showed a 0.88 probability of a splicing site loss.

We made an important and unexpected observation that a young female patient with a pathogenic variant in *BMPR1A* developed over 100 adenomatous polyps (as determined both by endoscopic and morphological examinations). Such a significant number of large adenomatous polyps is a major indication for proctocolectomy for young patients with FAP caused by the *APC* variants. In contrast, pathogenic *BMPR1A* variants mostly cause JPS, which is not an obligate pre-cancer syndrome. Thus, since the feasibility of prophylactic proctocolectomy for JPS is controversial, the optimal strategy for surgical treatment of such patients remains to be established and requires further investigation.

The remaining 7 patients had variants in different genes, including those responsible for the development of polyposis, but none of them were likely pathogenic or pathogenic.

We would like to highlight that employing different high throughput sequencing methods (gene panels or whole exome) would not have allowed us to detect the germline variants in our patients since they are located in the non-coding regions of the *APC* and *BMPR1A* genes. This further highlights the importance of WGS as a preferred method of analysis of patients who cannot be genetically diagnosed using Sanger sequencing and MLPA.

## Conclusions

In this study, using WGS, we identified pathogenic or likely pathogenic variants in the *APC* and *BMPR1A* genes in 3 out of 10 classic FAP patients for whom standard genetic methods of Sanger sequencing and MLPA failed to reveal the cause of disease. Thus, WGS may find its niche in the diagnosis of patients with over 100 adenomatous polyps.

## Data Availability

All relevant data is contained within the article. The original contributions presented in the study are included in the article/supplementary material, further inquiries can be directed to the corresponding author.
